# Vascular photobiomodulation in the treatment of children with temporomandibular disorders: Study protocol for a randomized, controlled, blind, clinical trial

**DOI:** 10.1097/MD.0000000000031228

**Published:** 2022-10-21

**Authors:** María Roxana Ferreira Sertaje, Marcela Leticia Leal Gonçalves, Andréa Oliver Gomes, Laura Hermida Bruno, Ana Laura Fossati, Natalia Osorio Viarengo, Elaine Marcilio Santos, Ana Paula Taboada Sobral, Raquel Agnelli Mesquita-Ferrari, Kristianne Porta Santos Fernandes, Anna Carolina Ratto Tempestini Horliana, Lara Jansiski Motta, Sandra Kalil Bussadori

**Affiliations:** a Universidad Católica del Uruguay (UCU), Montevideo, Uruguay; b Post Graduation Program in Biophotonics Applied to Health Sciences, Universidade Nove de Julho, São Paulo, SP, Brazil; c Postgraduation Program in Health and Environment, Universidade Metropolitana de Santos, Santos, SP, Brazil.

**Keywords:** ILIB, jaw range of motion, pain, photobiomodulation, temporomandibular disorder

## Abstract

This study aims to verify if the use of intravascular laser irradiation of blood (ILIB) influences the reduction of pain and increases the range of motion in opening and closing of the mouth in children and adolescents with temporomandibular disorders (TMD).

**Methods:**

This will be a blind, randomized, and controlled clinical trial, which will be carried out on children between 6 and 12 years of age who enter the Catholic University of Uruguay, Faculty of Health Sciences, Postgraduate School, for treatment. To be included, children must present temporomandibular disorders, based on the diagnostic criteria will be the Research Diagnostic Criteria for Temporomandibular Disorders (RDC/TMD). Forty-five participants will be randomized to three groups: Group 1—ILIB with 2 sessions of 20 minutes for 12 weeks (n = 15); Group 2—Placebo laser application with 2 sessions of 20 minutes for 12 weeks (n = 15); Group 3—Control with no treatment (n = 15). Irradiation will be performed by continuous and direct transcutaneous application to the radial artery, by means of a bracelet that inserts the laser beam. The laser to be used is infrared, power 100 mW ± 20%, wavelength 808 nm ± 10 nm, continuous application. RDC/TMD and pain evaluated through a visual analog scale will be the outcome measures.

**Discussion:**

Due to the low level of evidence, new studies are needed on the effect of ILIB in children with TMD.

## 1. Introduction

The American Dental Association has adopted the term temporomandibular disorder (TMD) to refer to all functional disturbances of the masticatory system.^[1]^ They represent a heterogeneous group of pathologies that affect the temporomandibular joint (TMJ), the masticatory muscles, or both, characterized by signs such as muscle and/or TMJ pain, TMJ sounds, and restriction or deviation of the opening trajectory from the mouth. The Research Diagnostic Criteria for Temporomandibular Disorders (RDC/TMD) is the reference standard among classification systems in this field of research.^[2]^ Many children diagnosed with TMD have adaptive physiological changes during growth and development, but if the diagnosis is late, already in an adult stage, it can become irreversible.

Low intensity laser therapy, or photobiomodulation (PBM), is capable of inducing a photobiological response within cells. The use of red and infrared light stimulates repair, relieves pain and reduces inflammation. The primary chromophores are identified as cytochrome c oxidase in mitochondria and calcium ion channels. Secondary effects of photon absorption include increases in ATP, in reactive oxygen species (ROS), in nitric oxide, and modulation of calcium levels. There is a dose regimen whereby low light levels have stimulating effects, while high light levels have inhibitory effects. Photobiomodulation is capable of upregulating antioxidant defenses and reducing oxidative stress. One of the most reproducible effects of photobiomodulation is an overall reduction in inflammation, which is particularly important for joint disorders.^[3]^

The most commonly used light sources for photobiomodulation are lasers (light amplification by stimulated emission of radiation) and LEDs (light-emitting diodes). Red phototherapy activates cell signaling pathways that modulate cell function. This light source is economical, consumable, portable, easy to operate, and can be combined with other treatments. It increases adenosine triphosphate (ATP),^[4,5]^ achieving a hemodynamic response of coupled oxygen delivery and blood volume,^[6]^ inducing positive effects on immunoglobulins, interferons and interleukins.^[4]^

Photobiomodulation regulates antioxidant defenses and reduces oxidative stress. The micro and macrovascular response accompanies the evidence shown by the systemic effects of intravascular laser irradiation of blood (ILIB).^[7]^ One of the most reproducible effects of photobiomodulation is an overall reduction in inflammation. It is also used to accelerate wound healing, collagen production, and modulation of the immune system.^[4,6]^ These characteristics are due to monochromaticity, the main property of laser light, that is, an extremely narrow spectral area (line), which makes it much easier to define and control the energy of laser light, besides being non invasive, low cost and safe.^[8,9]^

Therefore, the aim of this study is to verify if the use of intravascular laser irradiation of blood (ILIB) influences the reduction of pain and increases the range of motion in opening and closing in children and adolescents with TMD.

## 2. Methods

### 2.1. Study registration

This is the first version of a protocol that was registered in ClinicalTrials.gov, under the registration number: NCT05297604, first posted on 28 March, 2022 and last updated on 31 August, 2022. Available online at: https://clinicaltrials.gov/ct2/show/NCT05297604.

### 2.2. Type of study

**Figure 1.**
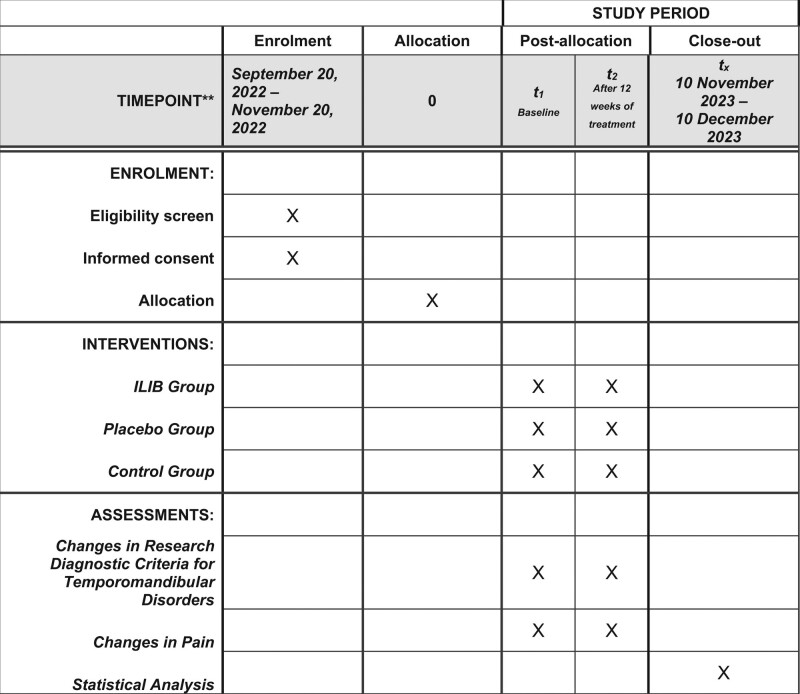
SPIRIT figure as recommended by 2013 SPIRIT statement. SPIRIT = Standard Protocol Items: Reccomendations for Interventional Trials.

This study is a protocol for a randomized, paralell group, blinded, controlled clinical trial. It is described according to the criteria established in the Standard Protocol Items: Reccomendations for Interventional Trials (SPIRIT) statement and Figure 1 is the SPIRIT figure.

### 2.3. Ethics committee

The study will follow the regulatory standards for research in human beings of the Research Ethics Committee of the Catholic University of Uruguay, under the process number 220824b. Participants must sign a written informed consent form after all aspects related to participation in the research have been clarified, in accordance with Decree No. 158/019, in force in the Eastern Republic of Uruguay. The children and adolescents will also sign an assent form. One of the researchers responsible for the procedures will explain the consente forms. The study will be carried out in accordance with the Declaration of Helsinki. The study participants will be recruited and treated at the Dental Diagnostic Clinic of the University Health Clinic, Graduate School of the Catholic University of Uruguay, from September 20, 2022 to May 20, 2023.

### 2.4. Participants

The study will include children between 6 and 12 years of age who enter the Catholic University of Uruguay, Faculty of Health Sciences, Postgraduate School, for treatment. Patients will already be in treatment for other conditions at the clinic, what will facilitate recruitment. It is believed that the need for treatment of TMD before major pain sympthoms will help to improve adherence to the trial.

#### 2.4.1. Inclusion criteria.

1. Healthy participants;
2. Children and adolescents diagnosed with TMD;
3. From 6 to 12 years old;
4. Have a complete dentition (except third molars);
5. Present deviation and/or mandibular deflection.

#### 2.4.2. Exclusion criteria.

1. Patients with craniofacial anomalies, genetic syndromes since this is due to the fact that people with anomalies of this type are more likely to suffer from TMD due to other factors associated with their malformation, which would be a confounding variable for this study.
2. Patients presenting occlusal changes; make use of any type of dental prosthesis; be undergoing orthodontic treatment or need orthognathic surgery or physical therapy; since it would alter the results of both diagnosis and treatment.
3. Patients undergoing cancer treatment: because it is necessary that patients do not have any condition that alters their general health, since this may cause a different response to the proposed treatment.
4. Patients who start using any type of medication during any phase of the study because it may cause a different response to the proposed treatment.
5. Patients with photosensitivity since it will not be possible to apply part of the treatment.

### 2.5. Evaluations

#### 2.5.1. Research Diagnostic Criteria for Temporomandibular Disorders.

The Research Diagnostic Criteria for Temporomandibular Disorders (RDC/TMD) will be used to determine TMD and assess participants. This is the primary outcome of the study and it will be assessed at baseline and after the 12 weeks of treatment. This evaluation consists of a clinical examination based on a detailed physical evaluation, checking the pattern of mouth opening, vertical extension of mandibular movement, TMJ sounds on palpation for vertical extension of movement, excursive mandibular movements, TMJ palpable sounds during right and left lateral excursion and protrusion.

The clinical diagnosis with RDC/TMD is divided into 3 groups: Group I, Muscle diagnoses (myofascial pain and myofascial pain with limited opening); Group II, Disc Displacement (displacement with reduction, displacement without reduction with limited opening, and displacement without reduction, without limited opening); Group III, Arthralgia, arthritis, osteoarthritis (arthralgia, TMJ osteoarthritis and TMJ osteoarthritis). The range of mandibular movement will be determined with the help of a digital caliper.^[10,11]^

The questionnaire that forms an integral part of the RDC/TMD is made up of 31 items that involve general health, oral health, history of facial pain, opening limitation, noise, habits, biting, tinnitus, illnesses in general, joint problems, headache, behavior, current economic, and social profile.

RDC/TMD reflects the interaction between clinical criteria and disabling features related to pain and psychological status, assigning from no diagnosis to a maximum of 5 (a Group I diagnosis + a Group II diagnosis + a Group III diagnosis) for each joint.

#### 2.5.2. Pain evaluation.

This is the secondary outcome of the study and it will be assessed at baseline and after the 12 weeks of treatment. Pain will be evaluated using a visual analog scale (VAS). It is a subjective scale to measure the intensity of pain, described by the patient. The child is asked to mark the intensity of the pain on a corresponding linear scale from 0 to 10, taking 0 without pain and 10 extreme pain. The point indicated by the patient is the intensity.^[10,12]^

### 2.6. Randomization

To randomly distribute the individuals in the experimental groups, a random sequence generator (https://www.randomizer.org/tutorial/) will be used. Opaque envelopes will be identified with each number and inside it a sheet containing the information of the corresponding experimental group will be inserted. The envelopes will be sealed and kept in a safe place until the time of the procedures. The generation of the sequence and the preparation of the envelopes will be performed by a person who is not involved in the study.

### 2.7. Interventions

#### 2.7.1. ILIB therapy protocol.

Participants will receive the ILIB treatment for 20 minutes. The laser to be used is infrared-red, with power of 100 mW ± 20%, wavelength 808 nm ± 10 nm, continuous application.^[13]^ These parameters were taken as a reference since they were presented in a previous article, in which it was considered safe and did not cause discomfort to the patients. The technique is not invasive, the point of irradiation is by continuous and direct transcutaneous application to the radial artery by means of a bracelet that inserts the laser beam. Two sessions of 20 minutes will be conducted per week, for 12 weeks.

Study participants will undergo 2 phases of collection. The first, called the Control Phase, in which the individuals are evaluated, and will remain for a week without any physical and/or dental intervention, followed by a new evaluation and the start of the second phase, called the Treatment Phase, in which individuals will be assigned to one of three research groups.

#### 2.7.2. Groups.

• Group 1 ILIB (n = 15): Participants will be subjected to ILIB protocol sessions. It is a noninvasive technique that consists of a low-intensity laser that is attached to a bracelet that has been developed so that the light beam is transported transcutaneously over the radial artery.^[12]^ They will receive 2 sessions per week for 12 weeks, and will be reassessed 1 month after the end of the proposed therapy.
• Placebo Group (n = 15): Participants will be treated in the same way as the ILIB group. The person in charge of the ILIB application will simulate the irradiation with the equipment kept off. So that the participant does not identify the group to which he/she/they belong, the device activation sound (beep) will be turned on at the time of application. They will receive 2 sessions per week for 12 consecutive weeks.
• Control Group (n = 15): Composed of individuals who will not receive any type of intervention. Before the procedure and 1 month after the end of therapy, pain will be assessed using the VAS and the opening and closing movement will be measured with a digital caliper.

### 2.9. Blinding

The clinical evaluations will be performed by examiners blinded to the groups to which each participant belongs. Unblinding is not permitted.

### 2.10. Sample size calculation

Sample calculations were performed using a significance level of 0.05 (which implies a type I error of 5% and will result in an analysis with a 95% confidence interval) and an absolute error of 5%. Considering a loss of 10%, a loss of 1.5 participants per group should be predicted, so 45 participants will be recruited, 15 per group.

### 2.11. Statistical analysis

The data will be tabulated and processed using the G Power version 3.1.9.2 program. The values will be tested for normality by the Kolmogorov–Smirnov test, and will be expressed as mean and standard deviation if Gauss curve is assumed. For the comparison between the groups, the T test will be carried out, considering a level of significance of 0.5% (*P* < .05). No loses greater than 5% of the sample are expected.

## 3. Discussion

Among the different methods of photobiomodulation, intravascular laser irradiation of blood (ILIB) has been shown to cause systemic effects. This technique has various analgesic and sedative effects. It is capable of facilitating blood circulation, causing widespread effects of intravenous blood irradiation on almost all systems. There is still no consensus in the literature on the parameters to be used in this form of treatment and on its efficacy in improving the clinical condition of growing and developing patients undergoing treatment with ILIB.^[4]^

Due to the high prevalence of children with TMD, we can treat this condition with ILIB, which is inexpensive, safe and has no side effects; there is also evidence on the systemic effect regarding the improvement in the body’s response in repair and inflammation, aiding in cases of decreased mandibular opening, for example.

Due to the low level of evidence, new studies are needed on the effect of ILIB in children with TMD.

## Author contributions

Conceive and design the study: MRFS, SKB, MLLG, RAMF, KPSF, ACRTH; will perform the experiment: MRFS, NOV, LHB, ANF; will analyze the data: EMS, LJM; write the paper: NOV, AOG, APTS, MLLG, SKB.

**Conceptualization:** María Roxana Ferreira Sertaje, Raquel Agnelli Mesquita-Ferrari, Kristianne Porta Santos Fernandes, Anna Carolina Ratto Tempestini Horliana, Sandra Kalil Bussadori.

**Formal analysis:** Elaine Marcilio Santos, Lara Jansiski Motta.

**Funding acquisition:** Sandra Kalil Bussadori.

**Investigation:** María Roxana Ferreira Sertaje, Laura Hermida Bruno, Ana Laura Fossati, Natalia Osorio Viarengo.

**Methodology:** María Roxana Ferreira Sertaje, Laura Hermida Bruno, Ana Laura Fossati, Natalia Osorio Viarengo.

**Resources:** Sandra Kalil Bussadori.

**Software:** Lara Jansiski Motta.

**Supervision:** Sandra Kalil Bussadori.

**Writing – original draft:** María Roxana Ferreira Sertaje, Andréa Oliver Gomes, Natalia Osorio Viarengo.

**Writing – review & editing:** Marcela Leticia Leal Gonçalves, Ana Paula Taboada Sobral, Anna Carolina Ratto Tempestini Horliana, Sandra Kalil Bussadori.
